# Dysfunctional CFTR Alters the Bactericidal Activity of Human Macrophages against *Pseudomonas aeruginosa*

**DOI:** 10.1371/journal.pone.0019970

**Published:** 2011-05-18

**Authors:** Paola Del Porto, Noemi Cifani, Simone Guarnieri, Enea Gino Di Domenico, Maria A. Mariggiò, Francesca Spadaro, Silvia Guglietta, Marco Anile, Federico Venuta, Serena Quattrucci, Fiorentina Ascenzioni

**Affiliations:** 1 Department of Biology and Biotechnology “Charles Darwin,” Sapienza University, Rome, Italy; 2 Department of Neuroscience and Imaging, Center of Excellence on Ageing (Ce.S.I), University “Gabriele d'Annunzio,” Chieti-Pescara, Italy; 3 Unit of Experimental Immunotherapy, Department of Cell Biology and Neurosciences, Istituto Superiore di Sanità, Rome, Italy; 4 Department of Thoracic Surgery, Sapienza University, Policlinico Umberto I, Rome, Italy; 5 Department of Pediatrics, Centro di Riferimento Fibrosi Cistica Regione Lazio, Sapienza University, Rome, Italy; French National Centre for Scientific Research - Université de Toulouse, France

## Abstract

Chronic inflammation of the lung, as a consequence of persistent bacterial infections by several opportunistic pathogens represents the main cause of mortality and morbidity in cystic fibrosis (CF) patients. Mechanisms leading to increased susceptibility to bacterial infections in CF are not completely known, although the involvement of cystic fibrosis transmembrane conductance regulator (CFTR) in microbicidal functions of macrophages is emerging. Tissue macrophages differentiate *in situ* from infiltrating monocytes, additionally, mature macrophages from different tissues, although having a number of common activities, exhibit variation in some molecular and cellular functions. In order to highlight possible intrinsic macrophage defects due to CFTR dysfunction, we have focused our attention on *in vitro* differentiated macrophages from human peripheral blood monocytes. Here we report on the contribution of CFTR in the bactericidal activity against *Pseudomonas aeruginosa* of monocyte derived human macrophages. At first, by real time PCR, immunofluorescence and patch clamp recordings we demonstrated that CFTR is expressed and is mainly localized to surface plasma membranes of human monocyte derived macrophages (MDM) where it acts as a cAMP-dependent chloride channel. Next, we evaluated the bactericidal activity of *P. aeruginosa* infected macrophages from healthy donors and CF patients by antibiotic protection assays. Our results demonstrate that control and CF macrophages do not differ in the phagocytic activity when infected with *P. aeruginosa*. Rather, although a reduction of intracellular live bacteria was detected in both non-CF and CF cells, the percentage of surviving bacteria was significantly higher in CF cells. These findings further support the role of CFTR in the fundamental functions of innate immune cells including eradication of bacterial infections by macrophages.

## Introduction

Cystic fibrosis is the most common genetic disorder affecting the Caucasian population. The disease is caused by mutations of the Cystic Fibrosis Transmembrane Conductance Regulator (CFTR) which encodes a c-AMP dependent chloride channel [1], [2]. Defective chloride secretion due to dysfunctions of CFTR results in the dehydratation of airway liquid leading to depletion of the periciliary layer and production of highly viscoelastic mucus which significantly impact mucociliary clearance and expectoration [3]. This combination of factors prevents the elimination of bacteria from the lung permitting bacterial infections to become established. Bacterial infections eventually lead to chronic inflammation, which accounts for the progressive tissue damage and ultimately to respiratory failure in CF [4]. Indeed CF lung inflammation is characterized by a sustained accumulation of neutrophils, high proteolitic activity and elevated levels of pro-inflammatory cytokines and chemokines [5]. Besides alteration of the mucociliary clearance system, CFTR mutations might affect other functions of bronchial epithelial cells including the internalization of *P. aeruginosa*, the release of inflammatory mediators, sphingolipid metabolism and transport of the redox buffer molecule GSH [6]–[9].

Recently, several reports suggest that CFTR might be of pivotal importance in the normal function of immune cells, such macrophages and neutrophils. For instance, Painter at al. have shown that CFTR expressed on phagolysosomes is crucial for the chlorination reactions involved in bacterial killing by human neutrophils [10], [11]. Studies in the CF knockout mice demonstrated that CFTR participates in phagosomal pH control of murine alveolar macrophages, thereby CFTR-deficient macrophages failed to acidify lysosomes and phagolysosomal compartments and displayed an altered bactericidal activity [12], [13]. Additionally, defects in the ROS mediated killing of *P. aeruginosa* by murine CFTR-deficient alveolar macrophages have been recently reported by Zhang et al [14]. This defect has been associated with the failure of infected macrophages to activate acid sphimgomyelinase and to release ceramide, thus preventing the formation of ceramide enriched membrane platforms that serve to cluster and activate NADPH oxidase [14].

Finally, a contribution of CFTR in the production of different cytokines by macrophages has been recently described. Bruscia et al. showed that in response to *P. aeruginosa* lipopolysaccharide (LPS), bronchoalveolar fluids from CF mice present significantly higher concentrations of macrophage derived pro-inflammatory cytokines such as IL-1α, IL-6, G-CSF and IL-8 as compared to wild type cells. Results from *in vitro* stimulation of alveolar and bone marrow derived macrophages with *P. aeruginosa* LPS confirmed the exuberant cytokine production in CF cells [15].

Although these data support the hypothesis that the abnormal macrophage activity, due to the lack of CFTR might be one of the causes of persistent bacterial infections and exuberant inflammatory responses in CF, at present the contribution of CFTR in the physiology of human macrophages is unknown. In order to define possible intrinsic macrophage defects due to CFTR deficiencies we have analyzed the bactericidal activity against *P. aeruginosa* of human monocyte derived macrophages from CF patients. To this aim we have first verified CFTR expression and functional activity in macrophages from healthy donors, subsequently, we have evaluated the capacity of CF macrophages to kill intracellular *P. aeruginosa*. Our results show for the first time that, macrophages derived from peripheral blood monocytes isolated from healthy donors express a functional CFTR and efficiently kill intracellular bacteria. Comparison of the bactericidal activity of control and CF macrophages revealed a significant increase in bacterial survival in cell carrying dysfunctional CFTR suggesting that CFTR, independently from tissue origin, directly contributes to microbicidal function of phagocytes.

## Materials and Methods

### Study subjects

Fifteen patients with CF (Regional Cystic Fibrosis Center, Sapienza University, Rome Italy), confirmed by positive sweat tests and genotyping (6 males, 9 females, median age 27) were enrolled in the study. Clinical and demographic characteristics of CF patients are reported in Table 1. Blood samples, for isolation of CD14^+^ cells, were collected when patients attended the clinic for routine evaluation. Informed written consent was obtained from all participants after approval of the study by the local ethics committee (Comitato Etico, Azienda Policlinico Umberto I, Rome, Italy; 21 June 2007). Blood samples from twelve, sex and age matched healthy donors were used as controls in bacterial infection assays. Additionally twelve healthy donor samples were used in RT-PCR experiments.

**Table 1:** Characteristics of CF patients.

Patient	Age	Sex	Genotype	Microbiology^a^	FEV1%
CF1	25	M	F508del/-	none	98%
CF2	34	F	F508del/F508del	*S.a.*	76%
CF3	16	F	F508del/F508del	none	87%
CF4	20	F	F508del/F508del	*S.a.*	49%
CF5	24	F	F508del/R1162X	*S.a,P.a.*	63%
CF6	34	F	F508del/L732X	*C.a., S.a.,P.a.*	33%
CF7	15	M	F508del/F508del	*A.t., S.m.*	39%
CF8	22	M	F508del/N1303K	*S.a., P.a.*	39%
CF9	23	M	F508del/F508del	*P.a.*	91%
CF10	33	F	F508del/F508del	*P.a.,P.f.*	49%
CF11	27	F	F508del/F508del	*P.a.*	28%
CF12	45	F	F508del/F508del	*P.a.*	44%
CF13	30	M	W1282X/W1282X	*P.a.*	43%
CF14	28	M	F508del/F508del	*S.a.*	66%
CF15	36	F	F508del/F508del	*S.a.*	76%

a *S.a.: Staphylococcus aureus; P.a.: Pseudomonas aeruginosa; C.a.: Candida albicans; A.t.: Aspergillus terreus; S.m.: Stenotrophomonas maltophilia; P.f.: Pseudomonas fluorescens;* - unknown;

### Isolation and differentiation of human monocytes

Peripheral blood mononuclear cells were isolated by density gradient centrifugation (Lympholyte, Cedarlane, Hornby, CA). CD14^+^ cells were purified from PBMC by positive selection with anti-CD14 mAb coupled to magnetic beads (Miltenyi Biotec, Bergisch Gladbach, Germany). The purity of CD14^+^ was routinely >90% as estimated by flow cytometry using FITC conjugated CD14 (BD Biosciences). CD14^+^ cells were differentiated for 7 days in RPMI 1640 (Gibco-BRL, Invitrogren Corporation Carlsbad, CA, USA) supplemented with 20% FCS and 100 ng/ml recombinant macrophage colony stimulating factor (MCSF; PeproTech Inch, Rocky Hill, NY, USA).

### RNA extraction and real time PCR

Total RNA was isolated from macrophages by Tryzol (Tryzol Reagent, Invitrogren Corporation Carlsbad, CA, USA) treated with DNase and purified using the RNeasy mini kit (Qiagen GmbH, Hilden, Germany) according to the manufacturer's instructions. cDNAs were obtained using Reverse Trascription System kit (Promega, Fitchburg, WI, USA) and random primers. Real time PCRs were done using SYBR Green PCR Master Mix (Applied Biosystem, Forster City, CA, USA) according to the suppliers' specification. The primers were as follow: for CFTR, rtCF-F1 5′-AAGCGTCATCAAAGCATGCC-3′(cDNA nt position 1686/1705) and RTCF-R1 (5′-TTGCTCGTTGACCTCCACTCA-3′ (cDNA nt position1775/1795); for the actin gene (used as the endogenous reference gene) rtbeta-ActF1 5′-GCCGGGACCTGACTGACT-3′ and rtbeta-ActR1 5′-TGGTGATGACCTGGCCGT-3′. Each sample was amplified in triplicates and CFTR mRNA level was determined by the ΔΔCt relative quantification method (7300 System SDS software, Applied Biosystem). Either parental monocytes or the alveolar epithelial cell line H441 (ATCC, HTB-174), which expresses low level of CFTR mRNA, were used as calibrators.

### Immunofluorescence and Confocal Laser Scanning Microscopy (CLSM) analyses

For CLSM analyses, 1×10^5^ *in vitro* differentiated macrophages were seeded in 24-well cluster plates on cover glasses (diameter, 12 mm) the day before analysis. Cells were fixed with paraformaldeyde 3% (PFA, 30 min 4°C) permeabilized with 0.5% Triton X-100 (10 min, room temperature) and then stained at 37°C with the anti-CFTR polyclonal antibody H-182 (Santa Cruz Biotechnology, Santa Cruz California), followed by Alexa Fluor 488-conjugated goat anti-rabbit IgG F(ab)2 (Molecular Probes, Eugene, OR). The cover glasses were extensively washed with PBS 1× and mounted on the microscope slide with Vectashield antifade mounting medium containing DAPI (Vector Laboratories, Burlingame, CA). To determine CFTR localization in the lysosomes, macrophages were first stained for CFTR using as secondary antibody Alexa Fluor-594 goat anti-rabbit IgG F(ab)2, and then they were labelled with the mouse anti human LAMP-1 antibody (Developmental Studies Hybridoma Bank, University of IOWA, IOWA City, IA) and the Alexa Fluor-488 F(ab)2 goat anti-mouse IgG. CLSM observations were performed with a Leica TCS SP2AOBS apparatus using excitation spectral laser lines 405, 488 and 594 nm and using a 63× oil immersion lens. Image acquisition and processing were achieved using the Leica Confocal Software (LCS) (Leica Lasertechnik, Heidelberg, Germany) and Adobe Photoshop software programs (Adobe system, Mountain View, CA). Signals from different fluorescent probes were taken in sequential scan settings, and co-localization was detected in yellow (pseudo-colour). At least 50 individual cells were analyzed for each staining condition. Isotype control antibodies were used in all confocal microscopy experiments to confirm the specificity of antibody staining.

### Electrophysiology recordings

All experiments were conducted at room temperature (22–24°C) using an Axopatch 200B patch clamp amplifier (Axon Instruments, Burlingame, CA, USA) and using the pCLAMP 9.0 and CLAMPFIT 9.0 as acquisition and data analyses programs respectively (Axon Instruments, Burlingame, CA, USA). Patch-clamp pipettes, obtained using borosilicate glass (Science Products GmbH, Hofheim, Germany), and a Heka puller (model PIP5, HEKA Elektronik, Lambrecht/Pfalz Germany), had resistances between 8–10 MOhms. Whole-cell recordings were performed according to the method previously described by Hamill et al. [16]. *In vitro* differentiated macrophage cells were plated on 35 mm Petri dishes at density of 1×10^5^ cells/dish, and, during the current recordings, bathed utilizing solutions in which Cl^−^ was the only possible permeable ion according to the protocol previously described for epithelial cells [17]. The pipette solution contained (in mM): 140 N-methyl D-glucamine; 40 HCl; 100 L-glutamic acid; 0.2 CaCl_2_; 2 MgCl_2_; 1 EGTA; 10 HEPES; and 2 ATP-Mg, pH 7.2. The bath solution contained (in mM): 140 N-methyl D-glucamine; 140 HCl; 2 CaCl_2_; 1 MgCl_2_; and 10 HEPES, pH 7.4. Cells were stimulated by the addition of a cAMP-activating cocktail (400 µM cAMP, 10 µM forskolin, 1 mM IBMX) to the pipette solution with voltage steps ranging from −110 to +110 mV for 200 ms with increments of 10 mV from a holding potential of −40 mV.

### Bactericidal assay

This assay was performed as described by Auriche C. et al. with minor modifications [18]. Briefly, the day before infection, macrophages were seeded in 48 well plates (1×10^5^ cells/well). *P. aeruginosa*, strain ATCC 27853, was grown over night in tryptic soy medium, harvested by centrifugation, washed twice in PBS 1× and once in serum-free RPMI 1640 medium before being re-suspended in RPMI 1640 supplemented with 10% FBS at ∼10^7^ CFU/ml. Based on preliminary experiments and previously published data macrophages were infected at a multiplicity of infection (MOI) of 30, i.e. 30 bacteria for one macrophage [12], [18]. Bacteria were brought in contact with macrophages by centrifugation (500 g for 10 min). The end of centrifugation was considered the starting point of infection which proceeded for 1 hr at 37°C in 5% CO_2_. After infection, the cells were gently washed with PBS 1× (three times) and incubated for 1 hr in culture medium containing gentamycin (400 mg/ml each) to kill the extracellular bacteria. The end of this step was codified as t0. Some wells were lysed to determine the number of intracellular bacteria at t0; others were incubated in antibiotic-free medium for additional four hours; samples were taken after 2 (t2) and 4 (t4) hours from t0.

Intracellular bacteria were counted by lysing the cells with 1% Triton X-100 in PBS 1× and plating serial dilutions of the lysates on PIA plates (Pseudomonas Isolation Agar). The fraction of internalized bacteria was determined with respect to the CFU used to infect the cells (the input), whereas bacterial survival was determined with respect to the CFU recovered at t0.

Each experimental section included the following controls: wells of uninfected macrophages; wells inoculated with bacteria only. Data were considered only when the following conditions were confirmed: lysates from un-infected macrophages did not revealed any bacterial colony on PIA plates; bacteria alone incubated for 1 hr with 400 mg/ml gentamycin were completely killed (<10 CFU left). Viability of macrophages after infection was determined by trypan blue counting in representative experiments and Annexin V staining.

### Statistical analysis

Mann-Whitney nonparametric test was used to investigate the significance of differences on bacteria counts between cases and controls at the different time-points analyzed. The same test was performed to evaluate differences on bacteria survival between cases and controls (at t2 and t4). *P* values less than 0.05 were considered statistically significant.

All the statistical procedures were performed by STATA 11 statistical package.

## Results

### CFTR expression in macrophages and precursor cells

**Figure 1:**
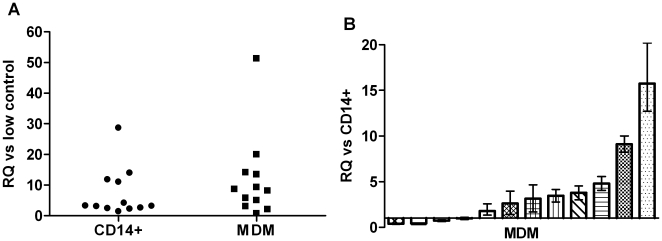
CFTR mRNA expression in human *in vitro* differentiated macrophages from non-CF donors. Panel A: Mean relative quantitiy (RQ) of CFTR mRNA in monocytes (CD14^+^) and monocyte derived macrophages (MDM) calibrated *versus* the H441 cells (low control); each symbol represents a single donor. Panel B: CFTR mRNA in MDM (mean RQ) calibrated *versus* parental monocytes. Each bar represents a single donor, whiskers above and below are the RQ-max and RQ-min, respectively.

As first, we wanted to assess whether the *CFTR* was expressed by *in vitro* differentiated macrophages and parental monocytes. For this purpose total RNA was extracted from both cell populations obtained by twelve healthy donors and CFTR mRNA was detected by real time PCR. The resulting data were analyzed by the relative quantification method using as calibrators either the low expressing control cells H441 or the parental monocytes (Fig. 1A and 1B). Both monocytes and macrophages showed CFTR expression, however the level of the CFTR transcript was higher in macrophages with respect to parental monocytes. Indeed, in most of the macrophage populations analyzed (8/12) we detected 2–15 fold increase of the CFTR mRNA in macrophages with respect to parental monocytes, in one sample we didn't observe significant variation and in 3 samples we observed a reduction down to half of that observed in monocytes (Fig. 1B). Overall these data demonstrate that *in vitro* differentiated macrophages express CFTR.

### Expression and functional activity of CFTR protein in differentiated macrophages

**Figure 2:**
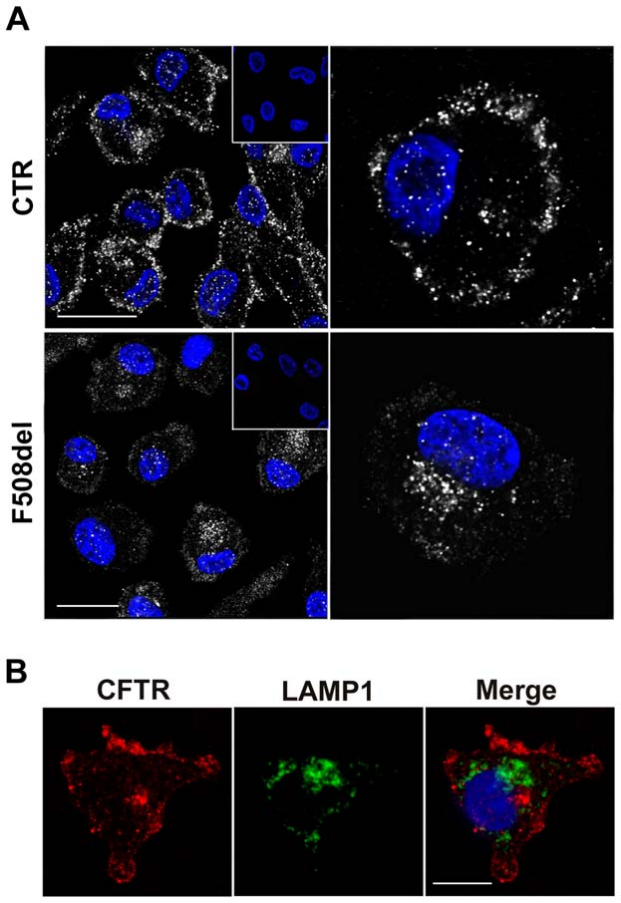
Confocal microscopy of the cellular localization of CFTR in control and F508del human *in vitro* differentiated macrophages. (A) Localization of CFTR in macrophages from non-CF (top row) and del508F homozygous CF individuals (bottom row). Permeabilized macrophages were stained with the polyclonal anti-CFTR antibody (H-182) and with Alexa Fluor 488-conjugated secondary antibody. Nuclei were counterstained with DAPI. Scale bars = 20 micron. Isotype negative controls are shown in the insets. (B) Permeabilized macrophages were stained with the anti-CFTR and the anti-LAMP1 antibodies; the secondary antibodies were Alexa Fluor-594 and Alexa Fluor-488 conjugated F(ab)2 IgG. Scale bar = 10 micron.

Having demonstrated the presence of CFTR mRNA in monocyte derived macrophages, we analyzed the expression of CFTR protein by immunofluorescence using the anti-CFTR antibody H-182 (Santa Cruz), raised against the first 182 aa of the protein. Examination of *in vitro* differentiated macrophages from control individuals by confocal microscopy showed that the majority of cells expressed CFTR which mainly localized at the plasma membranes or in their vicinity (Fig. 2A and 2B). Additionally, detectable intracellular staining was observed in a small percentage of the cells (Fig. 2A). By contrast, in CF macrophages homozygous for F508del mutation, the global immunofluorescence signal was much lower with respect to wild type (wt) cells and the brightest CFTR staining was found in the cytoplasm indicating a predominant intracellular localization of the protein (Fig. 2A).

In order to verify whether CFTR was also localized in some lysosomal vescicles of MDM, we double stained control MDM for CFTR and for the lysosomal-associated membrane protein 1 (LAMP1). Results from this analysis confirmed the plasma membrane localization of CFTR but they did not allow to demonstrate any co-localization of CFTR with the lysosomal marker as previously reported in murine lung macrophages (Fig. 2B).

**Figure 3:**
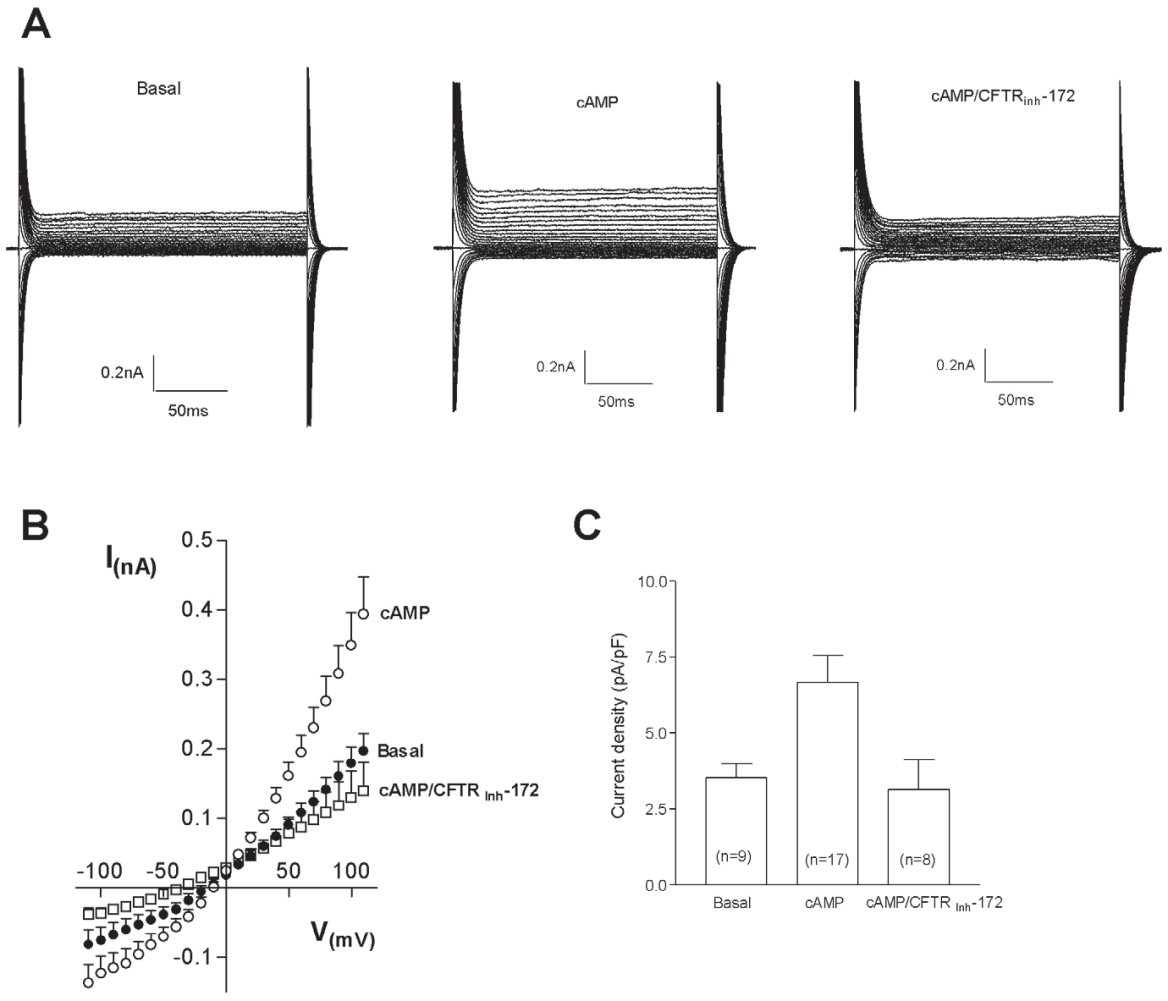
Whole-cell patch clamp of cAMP-evoked Cl^−^ current in human peripheral macrophages. (A) representative currents in basal condition (basal) or in response to the administration the cAMP-containing cocktail (cAMP), recorded on voltage-clamped macrophages. cAMP-evoked currents were blocked in the presence of the specific inhibitor CFTR_inh-172_ (10 µM), added to the bath solution (cAMP/CFTR_inh-172_). Currents were recorded in 200 ms voltage steps from −110 to +110 mV with 10 mV increments from a holding potential of −40 mV. (B) Averaged current/voltage relationship in basal conditions (basal, n = 9), in the presence of a cAMP-containing cocktail (cAMP, n = 16) or cAMP-containing cocktail plus 10 µM CFTR_inh-172_ (cAMP/CFTR_inh-172_, n = 7). (C) Current densities obtained at +110 mV in the three described experimental conditions, in parentheses the number of recorded cells are shown. Data reported in panel (B) and (C) are means±S.E.

To test if the antibody revealed protein was also functional, whole cell patch-clamp analyses were performed utilizing solutions in which chloride was the only mobile ion. This experimental protocol allowed to record currents from the entire CFTR channel population expressed on the plasma membrane of macrophages (basal; panel A Fig. 3). The presence of cAMP-containing cocktail, after 2–4 min of the establishment of the whole cell patch clamp configuration, stimulated a twofold increase in Cl^−^ currents with respect to the basal condition (cAMP, panel A Fig. 3). The treatment with the thiazolidinone CFTR_inh-172_, a selective blocker of the cystic fibrosis transmembrane conductance regulator, reverted the cAMP-evoked Cl^−^ currents (cAMP/CFTR_inh-172_, panel A Fig. 3). The graph in panel B of figure 3 shows the current/voltage relationship indicating that the currents appeared voltage independent with a chloride-selective reversal potential. The current density observed under basal conditions was 3,54±0,45 pA/pF, this value increased up to 6,67±0,88 pA/pF in cAMP-induced activation conditions, while in presence of CFTR_inh-172_ the obtained current density was 3,15±0,96 pA/pF, a value similar to that observed in basal conditions (panel C, Fig. 3).

### Bactericidal activity in wt and CF macrophages

Our data clearly demonstrate that CFTR is expressed and it is functionally active as a Cl^−^ channel in monocyte-derived macrophages isolated from healthy donors. Next, we determined the bactericidal activity of monocyte-derived macrophages from healthy donors (non-CF) and CF individuals against *P. aeruginosa*. A descriptive panel of the patients tested for macrophage bactericidal activity is reported in Table 1, in addition healthy donors matched for sex and age were included as controls. Overall the bactericidal activity was assayed in 15 CF samples and 12 non-CF controls.

The bactericidal activity of macrophages was assayed using the antibiotic protection method over a 4 hr time period. After infection, intracellular live bacteria were detected by the colony-forming unit (CFU) method at three time points: at the end of infection; two and four hours after infection. In order to ensure identical experimental conditions at least one non-CF and one CF sample were evaluated in each experimental section. The first data we have analysed was the number of intracellular live bacteria at the end of infection, which could be influenced by the phagocytic capacity of macrophages. The median number of live bacteria recovered from infected macrophages were 2108 and 1506 in HD and CF macrophages respectively with no significant differences between the two groups (*P* = 0,9611) suggesting that the phagocytic activity of these cells was very similar.

**Figure 4:**
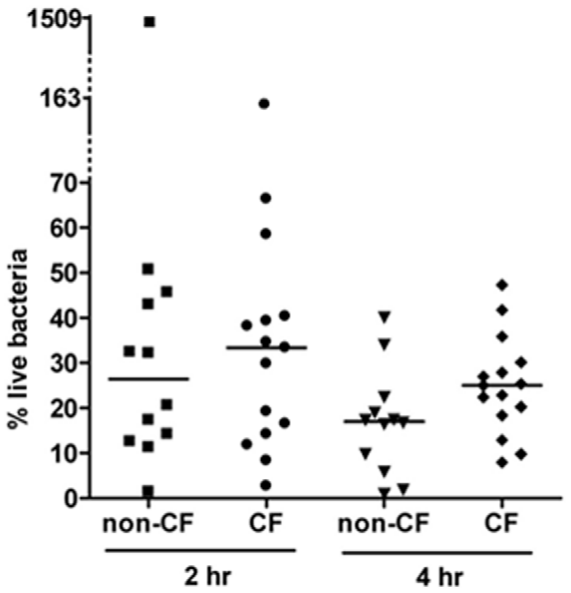
Surviving bacteria within human macrophages. Percentage of intracellular live bacteria rescued from *P. aeruginosa* infected macrophages two (2 hr) and four (4 hr) hours after infection. 100% refers to bacteria recovered at the end of infection (t0). Samples: non-CF, healthy donor macrophages (N = 12); CF, macrophages from cystic fibrosis patients (N = 15). Each symbol represents a single individual, the line is the median percentage of live bacteria.

**Figure 5:**
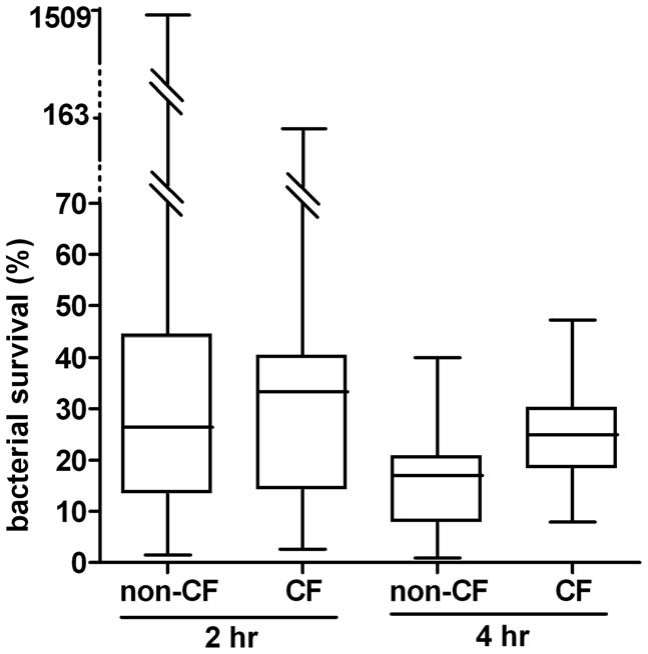
Summary data of live intracellular bacteria. The percentage of surviving bacteria two (t2) and four (t4) hours after infection with respect to live bacteria recovered at the end of infection (t0). Data are expressed as box plots representing, the 25 and 75 percentiles, median, minimal and maximal values. Statistical analysis: non-CF *vs* CF, *P* = 0,7697 and *P* = 0,0359 two and four hours after infection, respectively.

Next we determined the percentage of live bacteria two and four hours after infection with respect to the bacteria recovered at the end of infection. Two hours after infection, non-CF macrophages caused a rapid decline in the fraction of intracellular live bacteria down to a median percentage of survival of 26,4. Similarly, but to a lesser extent, CF macrophages reduced the percentage of intracellular live bacteria down to 33,34 (Fig. 4). Statistical evaluation of these results failed to reveal significant differences between the two groups at this time point. Only two macrophage samples were found to diverge from the majority: one non-CF sample showed an increase in the number of live bacteria two hours after infection of about two order of magnitude with respect to the other HD samples; similarly, at the same time point post-infection, one CF showed an increased number of live bacteria but by a factor of 10 (Fig. 4 and Fig. 5). However, both samples showed a subsequent reduction of intracellular live bacteria. Of note these samples have been analyzed in different experimental sections together with samples that behaved as the majority of macrophages. In the next time point analysed (four hours after infection) live bacteria recovered from non-CF macrophages were significantly less than those recovered from CF macrophages. Indeed the median percentage of live bacteria recovered from HD and CF macrophages was 16,90 and 25 respectively; (*P* = 0,0359). Although the deficit in the killing activity of CF versus non-CF macrophages was not as severe as previously reported for murine alveolar macrophages [12], [13] this result strongly suggests that CF macrophages do indeed display intrinsic deficiency of bactericidal activity. Furthermore, counting of viable macrophages and evaluation of apoptosis by annexinV staining (data not shown) failed to reveal differences in cell viability during the time course of the experiments excluding that the reduction in surviving bacteria was due to cell death. It has to be pointed out that the cellular model we have used for this analysis consists of macrophages which were not conditioned by the lung environment and thereby represent the best system to highlight possible intrinsic deficiencies of CF macrophages.

## Discussion

Chronic inflammation of the lung, as a consequence of persistent bacterial infections by several opportunistic pathogens represents the main cause of mortality and morbidity in CF patients [19]. At present the causes of the inability of CF patients to eradicate bacterial infections have been mainly ascribed to dysfunctions in the defence mechanisms mediated by airway epithelial cells. More recently, several studies in the murine model demonstrated that dysfunctional CFTR might alter the bactericidal activity of alveolar macrophages, further contributing to the poor control of bacterial growth in CF patients [12]–[14], [20]. In order to evaluate whether a similar defect affects human macrophages, we have compared the capacity of monocyte derived macrophages (MDM) from CF and control individuals to kill intracellular *P. aeruginosa*. The choice of this model was dictated by our primary goal i.e. analysis of possible intrinsic defects of macrophages which requires macrophagic cells unconditioned by the lung environment.

Due to the lack of available data, first we verified the expression and activity of CFTR in human macrophages by different techniques; i.e. RT-PCR, immunofluorescence, patch clamp recording. Our immunofluorescence localization data on MDM from healthy individuals demonstrated that wild type CFTR is predominantly localized at the plasma membranes or in the vicinity of them in the majority of MDM, with a small percentage of cells showing an intracellular localization. In contrast the same analysis on F508del homozygous cells revealed a strong reduction of plasma membrane staining compared to that observed in the cytoplasm. This latter finding might be explained by the elimination of misfolded CFTR by cellular quality control mechanisms. Indeed deletion of the F508 residue, which represents the most prevalent mutation in the CFTR, causes a temperature sensitive folding defect leading to protein degradation by the endoplasmic reticulum associated degradation machinery [21]. In addition our data are in agreement with previous reports showing that F508del CFTR protein mislocalizes from the apical membranes to the cytoplasm in primary airway and nasal epithelial cells [22], [23].

Consistently with plasma membrane localization of CFTR, whole-cell patch-clamp recordings from MDM from healthy individuals showed that the presence of Cl^−^ currents increased in the presence of an intracellular cAMP stimulation. As expected, the currents recorded were sensitive to CFTR_inh-172_, which has been reported to specifically block CFTR Cl^−^ conductance, supporting the results that the CFTR channels, not only are localized at plasma membrane, but are also functional [24]. Peak current density measured from human MDM during cAMP activation revealed lower values than those recorded by Di and collaborators from human alveolar macrophages [12]. This difference could be due to a different functional status of the analysed cell population; in addition, also different levels of CFTR on plasma membrane, due to different tissue origins, could be postulated.

It has been previously reported that CFTR is present not only at the plasma membranes but also within phagosomes, lysosomes and possibly other intracellular compartments in murine lung macrophages [12], [14]. Our results from double staining of permeabilized MDM with anti-CFTR and the anti-lysosomal marker LAMP1 did not support the expression of CFTR in lysosomes of human MDM. Differences between our data and those reported by Zhang and collaborators showing that CFTR localizes with some LAMP1 positive vesicles in freshly isolated lung macrophages might be explained either by differences in the cellular localization of CFTR in distinct species or in cells from different anatomical sites (periphery *vs* lung), or both. However, we cannot exclude that CFTR localization in lysosomal vesicles couldn't be detected by immunofluorescence due to low sensitivity of the method, indeed in the J774 murine macrophage cell line, immunoelectron microscopy was employed to co-localize CFTR and LAMP1 [12].

The role of CFTR in regulating bactericidal activity by macrophages was first demonstrated in 2006 in murine macrophages, this defect was observed in lung, but not peritoneal, macrophages and was related to the inability of CFTR*−/−* macrophages to maintain an acidic pH in the intralysosomal compartments [12]. Subsequent studies, by others as well as by the same group, produced contrasting results and the involvement of CFTR in lysosomal acidification is still debated [13], [25]–[28]. More recently measurements of ROS release, following *P. aeruginosa* infection, by WT and CFTR-deficient murine alveolar macrophages revealed a deficiency in ROS production and *P. aeruginosa* killing in CF macrophages. It has to be noted that Di and collaborators failed to reveal differences in ROS production by alveolar macrophages isolated from WT and CFTR-deficient mice [12]. Although the different experimental conditions might be responsible for these contradictions, whether CFTR activity has a direct role in phagosomal acidification and the mechanism/s responsible for macrophage dysfunction, are still opened questions. In this scenario, and due to the very few data on human macrophages, we have focused our attention on non-conditioned phagocytic cells, such as MDM, and studied the ability to kill *P. aeruginosa*. First, we determined whether differences could be found in the fraction of internalized bacteria at the end of infection between WT and CF macrophages. Statistical analysis of our data failed to reveal any significant difference suggesting that the phagocytic activity of monocyte derived macrophages is not affected by CFTR. Next, we determined the outcome of the intracellular bacteria over a period of 4 hour after infection. In the first 2 hours after infection, the fraction of live bacteria decreased at similar extend in WT and CF macrophages. On the contrary 4 hours after infection, although live bacteria continued to decrease, those recovered from CF macrophages were significantly more than from control cells. Thereby, for the first time to the best of our knowledge, we show that the bactericidal activity of human macrophages is indeed affected by CFTR-deficiency. Defective clearance of apoptotic cells has been previously reported in human MDM when exposed to the aqueous sol fraction of sputum recovered from CF patients. This impairment has been associated with the cleavage of phosphatidylserine receptor in a neutrophil elastase dependent manner [29]. Our data highlights a novel dysfunction of human CF MDM which, since it cannot be ascribed to conditioning by CF lung environment, reflects a primary defect of CF MDM. It is interesting to note that the observed defect in the bactericidal activity of human CF MDM was less profound than that previously demonstrated in murine CF alveolar macrophages. A possible explanation for this finding is the existence of multiple bactericidal mechanisms employed by human MDM to kill *P. aeruginosa* whose, only a part might be affected by CFTR deficiency as previously reported for human neutrophils [11]. Two strong clinical correlations have been identified in CF: i) a positive correlation between persistent *P. aeruginosa* infection and worst prognosis for the patients ii) a marked adaptation of the bacteria to the CF lung by a number of changes including loss of bacteria motility, altered antibiotic susceptibilities and metabolic shift (decreased oxygen consumption and increased nitrate utilization) [30]–[33]. Since our data were obtained with a *P. aeruginosa* strain, that might not recapitulate the phenotypes of chronic isolates it is reasonable to surmise that the observed deficit of bactericidal activity of CF macrophages could be more pronounced when challenged with late clinical isolates. It is generally accepted that phagocytic cells use a combination of oxidative and non-oxidative mechanisms to defend against a great variety of engulfed microorganisms [34]. Having demonstrated that the microbicidal activity of CF macrophages is significantly reduced four hours after infection but not at the previous time point (two hours), it might be hypothesised that multiple bactericidal mechanisms operating with different kinetics are differently affected by CFTR activity. Experiments are underway to analyse the molecular pathways leading to bacteria killing in WT and CF MDM.
